# Activation of the VEGF-A/ERK/PLA2 Axis Mediates Early Retinal Endothelial Cell Damage Induced by High Glucose: New Insight from an In Vitro Model of Diabetic Retinopathy

**DOI:** 10.3390/ijms21207528

**Published:** 2020-10-13

**Authors:** Giovanni Giurdanella, Gabriella Lupo, Florinda Gennuso, Federica Conti, Debora Lo Furno, Giuliana Mannino, Carmelina Daniela Anfuso, Filippo Drago, Salvatore Salomone, Claudio Bucolo

**Affiliations:** Department of Biomedical and Biotechnological Sciences, School of Medicine, University of Catania, via S.Sofia 97, 95123 Catania, Italy; g.giurdanella@unict.it (G.G.); gabriella.lupo@unict.it (G.L.); florigennuso@gmail.com (F.G.); conti.federica1@hotmail.it (F.C.); lofurno@unict.it (D.L.F.); juliana81@tiscali.it (G.M.); daniela.anfuso@unict.it (C.D.A.); f.drago@unict.it (F.D.); bucocla@unict.it (C.B.)

**Keywords:** phospholipase A2, retinal endothelium, VEGF-A, Aflibercept, high glucose

## Abstract

Early blood retinal barrier (BRB) dysfunction induced by hyperglycemia was related to increased pro-inflammatory activity of phospholipase A2 (PLA2) and the upregulation of vascular endothelial growth factor A (VEGF-A). Here, we tested the role of VEGF-A in high glucose (HG)-induced damage of human retinal endothelial cells (HRECs) mediated by Ca++-dependent (cPLA2) and Ca++-independent (iPLA2) PLA2s. HRECs were treated with normal glucose (5 mM, NG) or high glucose (25 mM, HG) for 48 h with or without the VEGF-trap Aflibercept (Afl, 40 µg/mL), the cPLA2 inhibitor arachidonoyl trifluoromethyl ketone (AACOCF3; 15 µM), the iPLA2 inhibitor bromoenol lactone (BEL; 5 µM), or VEGF-A (80 ng/mL). Both Afl and AACOCF3 prevented HG-induced damage (MTT and LDH release), impairment of angiogenic potential (tube-formation), and expression of VEGF-A mRNA. Furthermore, Afl counteracted HG-induced increase of phospho-ERK and phospho-cPLA2 (immunoblot). VEGF-A in HG-medium increased glucose toxicity, through upregulation of phospho-ERK, phospho-cPLA2, and iPLA2 (about 55%, 45%, and 50%, respectively); immunocytochemistry confirmed the activation of these proteins. cPLA2 knockdown by siRNA entirely prevented cell damage induced by HG or by HG plus VEGF-A, while iPLA2 knockdown produced a milder protective effect. These data indicate that VEGF-A mediates the early glucose-induced damage in retinal endothelium through the involvement of ERK1/2/PLA2 axis activation.

## 1. Introduction

Blood retinal barrier (BRB) dysfunction represents an early pathological event in diabetic retinopathy (DR) [1,2]. Hyperglycemia impairs endothelial cells (ECs), the main component of BRB, through the activation of inflammatory processes that lead to a reduction of the vasodilation and an increase of vascular leakage [3,4]. Pathophysiology of DR is considered a multifactorial process, where the activation of several interacting pathways occurs, leading to production of reactive oxygen species (ROS), pro-inflammatory mediators, and vascular endothelial growth factor (VEGF-A) [5,6,7,8]. Several cellular and molecular mechanisms underlying hyperglycemia-induced microvascular damage are well known, but the research in the field could still provide novel therapeutic targets and related therapeutics approaches. Phospholipase A2 (PLA2) plays an important role in cellular injury as it mediates inflammatory processes through the mobilization of arachidonic acid from membrane phospholipids [9,10,11]. Increased hydrolysis of membrane phospholipids by PLA2 has been related to an increased permeability of plasma membrane, ROS-induced damage, and apoptosis in response to pro-inflammatory stimuli [12,13,14,15]. PLA2 is activated in an in vivo model of diabetic retinopathy, involving glucose-induced injury and death of retinal pericytes and endothelium, with subsequent breakdown of the blood–retinal barrier [16]. Moreover, systemic inhibition of lipoprotein-associated PLA2 (Lp-PLA2) is able to effectively prevent diabetes-mediated BRB dysfunction in rats [17]. PLA2 mediates glucose-induced breakdown of BRB and pericyte damage, by upregulating cyclooxygenase-2 (COX-2), prostaglandin (PG) synthesis, VEGF-A, intercellular adhesion molecule-1 (ICAM-1), and tumor necrosis factor-alpha (TNF-α) in vitro [18,19]. PLA2 represents a heterogeneous superfamily of enzymes that hydrolyze the sn-2 ester bond in phospholipids, releasing free fatty acids and lysophospholipids [10,20]. PLA2 isoforms were classified into three categories; the secretory PLA2 (sPLA2) and the cytosolic PLA2 (cPLA2s), both requiring millimolar and micromolar levels of Ca^2+^, respectively, for their activities; and Ca^2+^-independent PLA2s (iPLA2s) [20]. Following pro-inflammatory stimuli, cPLA2 mediates arachidonic acid (AA) release through a Ca^2+^-dependent mechanism of translocation from cytosol to perinuclear membranes [21]. Moreover, cPLA2 activity is enhanced following phosphorylation of Ser-505 in the catalytic domain by ERK1/2, a mitogen-activated protein kinase (MAPK), and Ser-727 by other MAPKs [21,22]. Regulation of iPLA2 activity involves multiple mechanisms, including the ATP-mediated stabilization of the catalytic site, which is independent of enzyme phosphorylation, as indicated by the consensus nucleotide-binding motif (GGGVKG) [23,24,25]. Moreover, iPLA2 gene expression can be induced by different stress-related transcriptional factors, as indicated by the presence of numerous putative consensus sequences in its highly conserved promoter region [25]. Under pro-inflammatory stimuli, the sterol regulatory element (SRE) is recognized by the active form of its correspondent binding proteins (SREBPs), which leads to the up-regulation of iPLA2β transcription and protein expression [26,27,28]. Activation of ERK1/2 triggers cPLA2 phosphorylation and the subsequent increase of its enzymatic activity [29,30,31]. VEGF-A induces cPLA2 in human retinal microvascular endothelial cells, during hypoxia-induced retinal neovascularization by the involvement of VEGFR-2 and VEGFR-1 receptors [32,33]. Despite the highest levels of serum VEGF-A being found among proliferative DR patients, a significant increase in serum VEGF-A is also described in the early stage of DR, i.e., in non-proliferative DR patients, as compared to type 2 diabetes mellitus subjects without retinopathy [34]. VEGF-A stimulates the cPLA2-mediated release of arachidonic acid by the activation of p42/p44 MAP kinases in vitro, indicating a close molecular crosstalk between VEGF-A signaling and PLA2 [35]. Furthermore, inhibition of iPLA2-VIA significantly reduces both the spontaneous and the VEGF-induced proliferation and migration of the human RPE cell line [36]. In this study, we tested the hypothesis that VEGF-A impairs human retinal endothelial cells (HRECs) subjected to high glucose through Ca++-dependent (cPLA2) and Ca++-independent (iPLA2) phospholipase A2. In this respect, HRECs were treated with a high concentration of glucose, to mimic the early stage of DR; thereafter, the effects of the VEGF-trap Aflibercept, PLA2 blockade by chemical agents or knock down by siRNA, and exogenous VEGF-A were evaluated.

## 2. Results

### 2.1. Effects of Aflibercept and PLA2 Inhibitors on Retinal Endothelial Cell Damage Induced by HG

**Figure 1:**
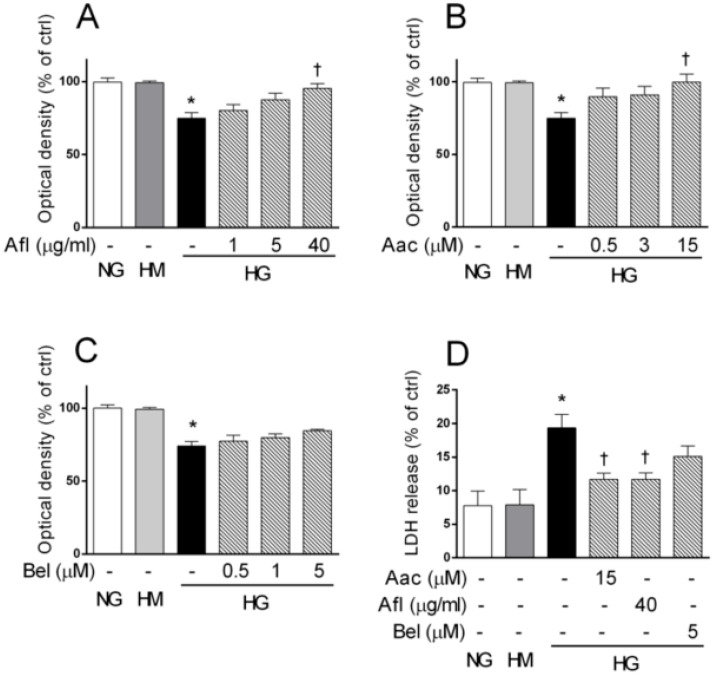
Aflibercept and the cPLA2 inhibitor AACOCF3 reduced cell damage in human retinal endothelial cells (HRECs) treated with high glucose (HG). HRECs were treated for 48 h with normal glucose (NG, 5 mM), high mannitol (HM, 25 mM) or high glucose (HG, 25 mM), alone or supplemented with increasing amounts of Aflibercept (Afl, 1, 5, and 40 µg/mL), the cPLA2 inhibitor AACOCF3 (Aac, 0.5, 3, and 15 µM) or the iPLA2 inhibitor (Bel, 0.5, 1, and 5 µM). After the treatments, cells were assessed for viability (MTT assay, panels **A**–**C**). Cells treated with maximal drug concentrations were also assessed in a cytotoxicity test (LDH release, panel **D**). Values are expressed as a mean ± SEM of three independent experiments, each run in triplicate. * *p* < 0.05 vs. CTRL; † *p* < 0.05 vs. HG alone. One-way ANOVA, followed by Tukey’s test.

It has been previously reported (i) that high glucose-induced toxicity in HRECs depends on PLA2 activity [19,29], and (ii) that the induction of cPLA2 is mediated by VEGF-A signaling (II) [35,37]. Thus, we first compared the capability of either the VEGF-trap Aflibercept or the inhibitors of cPLA2 and iPLA2 activity (AACOCF3 and BEL respectively) to counteract the cell damage induced by high glucose in HRECs. To mimic the insult of retinal endothelium occurring in diabetes, HRECs were treated with 25 mM glucose (high glucose, HG) for 48 h while 25 mM mannitol (high mannitol, HM,) was used as osmolarity control. High glucose damage was evaluated in terms of cell viability (MTT assay) and cytotoxicity (cell membrane permeability, LDH release) in comparison to control HRECs (5 mM glucose, NG). As expected, HG decreased by about 25% the optical density in MTT assay (reduced HRECs viability) while no changes were observed in HM-treated cells (Figure 1).

Aflibercept or AACOCF3 restored cell viability in a concentration-dependent manner, that reached statistical significance at concentrations of 40 μg/mL and 15 μM, respectively (Figure 1, panels A and B). A similar trend was observed with the iPLA2 inhibitor BEL, though not statistically significant (Figure 1 panel C). Both Aflibercept and PLA2 inhibitors were tested up to high, but still subtoxic concentrations; 48 h exposure to higher amounts resulted indeed in toxic effects (data not shown). As a consequence of HG-induced toxicity, the permeability of cell membrane increased, as indicated by a 2.4–fold increase in LDH activity measured in culture medium (Figure 1 panel D). No significant change in LDH release was detected in HRECs treated with HM. Increased release of LDH in HG-treated HRECs was largely prevented by 40 μg/mL Aflibercept or 15 μM AACOCF3 (*p* < 0.05), consistent with previous observations. Again, the effect of 5 μM BEL, if any, was less pronounced.

Overall, these data suggested the involvement of VEGF-A/PLA2 axis in HG-mediated damage in HRECs. Moreover, pharmacological blockade of VEGF-A or PLA2 exerted a similar effect in counteracting glucose-induced cell damage. 

### 2.2. Effect of Aflibercept and the cPLA2 Inhibitor AACOCF3 on Tube-Like Structure Formation in HRECs Impaired by HG

**Figure 2:**
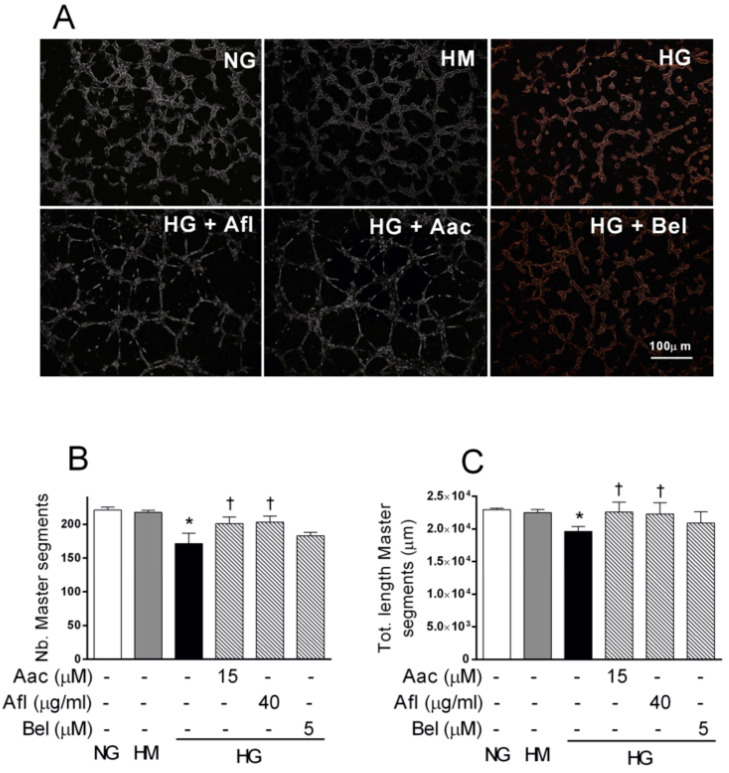
Aflibercept and cPLA2 inhibitor AACOCF3 increased the tube-like structures assessed by tube formation assays in human retinal endothelial cells (HRECs) treated with high glucose (HG). HRECs were seeded into the 96-well plate coated with Matrigel at a density of 1.5 × 10^4^/well in presence of normal glucose (NG, 5 mM), or high mannitol (HM, 25 mM) or high glucose (HG, 25 mM) alone or supplemented with Aflibercept (Afl, 40 µg/mL), of cPLA2 inhibitor (Aac, 15 µM) or iPLA2 inhibitor (Bel, 5 µM). After 4 h, tube-like structures were photographed and the images were analyzed with Image J software. Panel (**A**) shows representative photographs of tube-like structures. Quantitative analysis of total number and length of tube-like structures are shown in panels (**B**) and (**C**), respectively. Values are expressed as a mean ± SEM of three independent experiments, each run in triplicate. * *p* < 0.05 vs. CTRL; † *p* < 0.05 vs. HG alone. One-way ANOVA, followed by Tukey’s test.

We further investigated the role of VEGF-A/PLA2 axis blockade by testing the tube formation capability of HRECs challenged by HG, in presence of Aflibercept or PLA2 inhibitors. Tube formation assay was carried out in Matrigel, as previously described [29,38]. As shown in Figure 2 panel A, HG impaired the formation of tube-like structures in HRECs, as indicated by the significant reduction of master segment numbers and length (by about 25%, *p* < 0.05). 

The HREC capacity to form tubes was unaffected by HM. Treatment with 40 μg/mL Aflibercept or 15 μM of AACOCF3 significantly prevented the reduction of tube-like structures induced by HG. In fact, analysis of the tube-like structures showed number and length of master segments similar to controls (Figure 2, panels B and C, respectively). Co-treatment with HG and 5 μM of BEL barely affected the tube formation capability impaired by HG. These data showed that blockade of VEGF-A/PLA2 axis prevented the impairment of the tube-formation capacity induced by HG in HRECs.

### 2.3. Effects of Aflibercept and PLA2 Inhibitors on VEGF-A/PLA2 Axis Activation in HRECs Stimulated by HG

**Figure 3:**
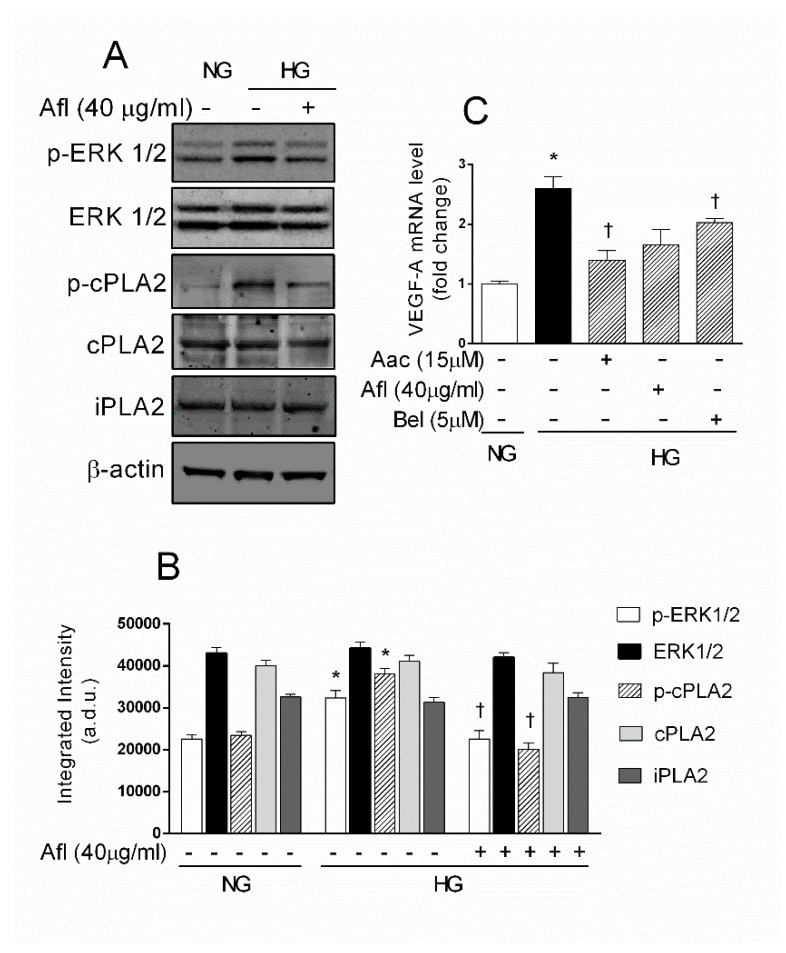
Aflibercept reduced phospho-cPLA2, phospho-ERK 1/2 and VEGF-A levels in human retinal endothelial cells (HRECs) treated with high glucose (HG). Cells were treated for 48 h with normal glucose (NG, 5 mM), high glucose (HG, 25 mM) alone or supplemented with Aflibercept (Afl, 40 µg/mL) or the cPLA2 inhibitor (Aac 15 µM). Panel (**A**) immunoblot analysis of whole-cell lysates from treated HRECs using antibodies against phospho ERK 1/2, total ERK1/2, phospho-cPLA2, total cPLA2 and iPLA2. The blot was probed with anti β-actin antibody for verify equal loading of 30 µg protein per lane. Panel (**B**) Densitometry analysis of immunoblot indicating protein quantification of each band (in arbitrary densitometry unit, a.d.u.), carried out with the Image J program. Bar graphs represent the means ± SEM from three independent experiments. Panel (**C**) RT-qPCR analysis of VEGF-A mRNA extracted from treated HRECs. Each bar represents the means ± SEM from three independent experiments. * *p* < 0.05 vs. Control (NG); † *p* < 0.05 vs. HG. One-way ANOVA, followed by Tukey’s test.

Activation of the ERK/cPLA2/COX-2/PGE2 axis represents an early event after exposure to HG, mediating the damage to retinal endothelium. This inflammatory pathway was related to increased VEGF-A expression triggered by glucose in retinal endothelial cells and pericytes [19,29]. We therefore tested the effect of the VEGF-trap Aflibercept in HG-mediated activation of ERK 1/2 and cPLA2 in HRECs. The amount of the active phosphorylated form of ERK1/2 and cPLA2 was assessed by immunoblot (Figure 3, panel A). 

As shown in Figure 3 panel B, HG treatment caused a significant increase in the level of both phospho-ERK1/2 (about 1.2-fold) and phospho-cPLA2 (about 2.3-fold). Increased phosphorylation levels of cPLA2 are indicative of an increase in its activity and thereby in the production of arachidonic acid available for prostaglandin synthesis. No changes were observed in phospho-ERK1/2 and phospho-cPLA2 protein levels of HRECs treated with HM [29]. Co-treatment with HG supplemented with 40 μg Aflibercept produced a decrease of both phospho-ERK1/2 and phospho-cPLA2. Moreover, this treatment did not trigger any significant change in the total amount of iPLA2 protein levels, confirming previous data. Since both endothelial cells and pericytes exposed to HG increase VEGF-A expression [19,29], we verified whether the protective effect of Aflibercept and PLA2 inhibitors may be related to inhibition of VEGF-A. Quantitative RT-qPCR showed a significant increase of about 1.5-fold in mRNA levels of VEGF-A in HRECs treated with HG compared to control (Figure 3 panel C). Treatment with 15 μM AACOCF3, 40 μg/mL Aflibercept, or 5 μM of BEL prevented the HG-induced upregulation of VEGF-A. These data indicate that HG elicit cell damage in HRECs through cPLA2 activation mediate by VEGF-A.

### 2.4. Effects of Exogenous VEGF-A in HG-Stimulated HREC

**Figure 4:**
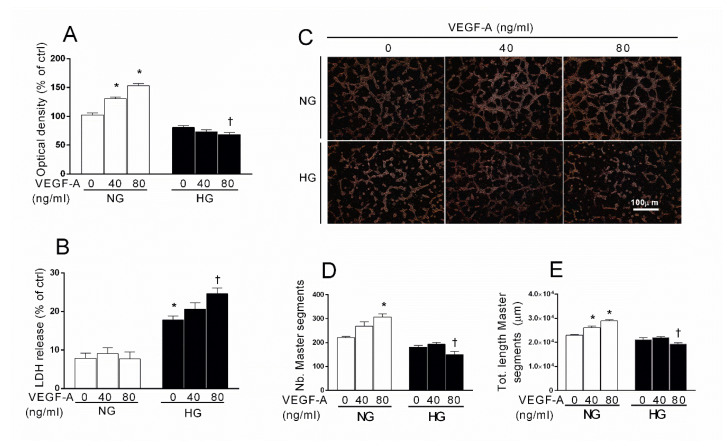
Exogenous VEGF-A increase damage in human retinal endothelial cells (HRECs) treated with high glucose (HG). Cells were treated for 48 h with normal glucose (NG, 5 mM), with or without VEGF-A (40 or 80 ng/mL) or with high glucose (HG, 25 mM) with or without VEGF-A (40 or 80 ng/mL). After the treatments, cells were subjected to cell viability tests (MTT assays, panel **A**) and to cytotoxicity tests (LDH release, panel **B**). HRECs were seeded into the 96-well plate coated with Matrigel at a density of 1.5 × 10^4^/well in presence of treatment media. After 16 h, tube-like structures were photographed and the images were analyzed with Image J software. Panel (**C**) shows representative photographs of tube-like structures. Quantitative analysis of total number and length of tube-like structures are shown in panels (**D**) and (**E**), respectively. Bar graphs represent the means ± SEM from three independent experiments. * *p* < 0.05 vs. Control (NG); † *p* <0.05 vs. HG. One-way ANOVA, followed by Tukey’s test.

Both Aflibercept and the cPLA2 inhibitor AACOCF3 appeared to prevent HG-induced damage in HRECs by counteracting the VEGF-A/ERK1/2/cPLA2 cascade. VEGF-A is known as the key physiological inducer of endothelial proliferation and vasculogenesis; yet, as mentioned above, a VEGF-trap such as Aflibercept, paradoxically increased the angiogenic potential (tube formation assay) in the presence of high glucose. Thus, in order to understand the role of VEGF-A in the early endothelial injury induced by glucose, we tested the hypothesis that VEGF-A might show a synergism and/or trigger additive mechanisms that exacerbate the aversive role of PLA2, activated by high glucose. To this end, we tested the effect of the treatment with 40 and 80 ng/mL of exogenous VEGF-A in presence of HG or NG for 48 h. As shown in Figure 4 panel A, exposure to exogenous VEGF-A in NG produced a significant increase of about 25 and 50% (at concentration of 40 and 80 ng/mL of VEGF-A, respectively, *p* < 0.05) in MTT reduction by HRECs, presumably as a result of cell proliferation.

Interestingly, the co-treatment with VEGF-A and HG slightly reduced MTT reduction, presumably because of cell damage and reduced cell viability (as also testified by LDH assay, see below), that became significant at the concentration of 80 ng/mL in comparison to HG-treated control (VEGF-A untreated) cells. No changes were observed in LDH release of HRECs treated with NG and VEGF-A (Figure 4 panel B). However, treatments with HG and increasing concentrations of VEGF-A increased LDH activity in culture medium in a dose-dependent manner: we observed a significant increase in LDH release of about 25% in HRECs treated with HG plus 80 ng/mL of VEGF-A in comparison to HG alone (*p* < 0.05). Moreover, we compared the impact of exogenous VEGF-A on the angiogenic potential of HREC in NG and HG, using the Matrigel tube formation assay. As shown in Figure 4 panel C, in NG VEGF-A stimulated angiogenesis in a concentration-dependent manner, as indicated by the increased formation of the cell network. Analysis of the cell networks showed, in fact, that VEGF-A increased both the number and the length of tube-like structure compared to the control. As expected, HG produced a reduction of cell network formation both in terms of number and length of tube-like structures, as a consequence of HG-induced endothelial injury. However, in contrast with NG, in HG VEGF-A did not trigger angiogenesis but slightly reduced the formation of tube-like structures. Both the number and the length of tube-like structures were significantly decreased in HRECs treated with 80 ng/mL of VEGF-A in comparison of HG-treated cells. These data support the hypothesis that, at least in our in vitro model, VEGF-A induces endothelial injury in the presence of HG.

### 2.5. Activation of ERK1/2/cPLA2 Pathway and iPLA Expression by VEGF-A in Presence of HG in HREC

**Figure 5:**
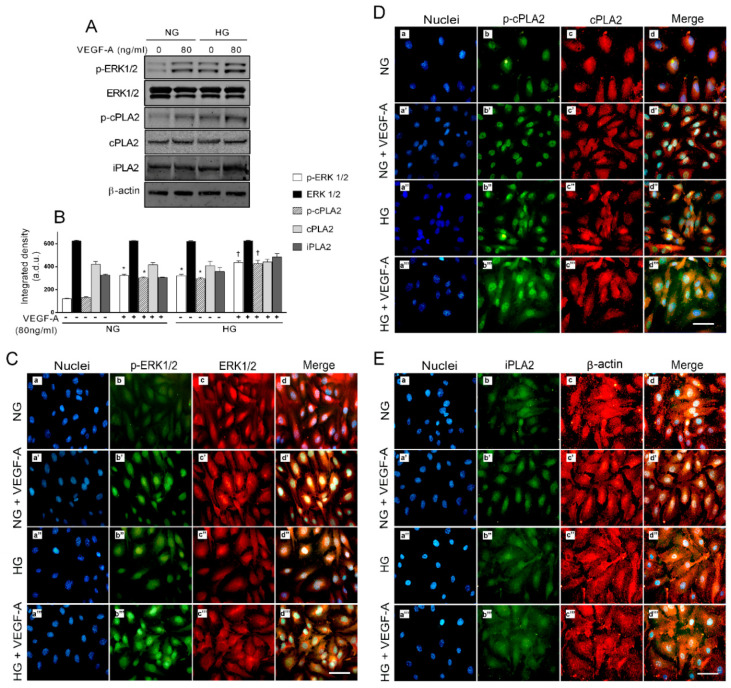
Exogenous VEGF-A exacerbated high glucose (HG)-induced activation of ERK/PLA2 axis in human retinal endothelial cells (HRECs). Panel (**A**): immunoblot analysis of HREC whole-cell lysates, using antibodies against phospho-ERK1/2 (p-ERK1/2), total ERK1/2, phospho-cPLA2 (p-cPLA2), total cPLA2 and iPLA2. The blot was probed with anti β-actin antibody to verify equal loading of 30 µg proteins per lane. Panel (**B**): densitometric analysis of immunoblot indicating protein quantification of each band (in arbitrary densitometry unit, a.d.u.), carried out with the Image J program. Panel (**C**): immunocytochemical staining for p-ERK 1/2 (green fluorescence) and ERK 1/2 (red fluorescence) in HRECs grown in normal glucose (NG, 5 mM; a, b, c, d), in normal glucose plus 80 ng/mL of VEGF-A (a’, b’, c’, d’), in high glucose (HG, 25 mM; a’’, b’’, c’’, d’’) or in high glucose supplemented with 80 ng/mL of VEGF-A (HG + VEGF-A; a’’’, b’’’, c’’’, d’’’). Blue fluorescence indicates DAPI staining of cell nuclei. Merged pictures are shown in the fourth column (d, d’, d’’, and d’’’). In control HRECs, green fluorescence was almost undetectable, either in the cytoplasm or in the nucleus. HG-culture conditions induced an increase in green fluorescence (indicating p-ERK1/2 activation), particularly in the nuclear region (b’’’ vs. b’ and b’’ vs. b, respectively). Panel (**D**): immunocytochemical staining for p-cPLA2 (green fluorescence) and cPLA2 (red fluorescence) in HRECs grown in normal glucose (NG, 5 mM; a, b, c, d), in normal glucose plus 80 ng/mL of VEGF-A (a’, b’, c’, d’), in high glucose (HG, 25 mM; a’’, b’’, c’’, d’’) or in HG supplemented with 80 ng/mL of VEGF-A (HG + VEGF-A; a’’’, b’’’, c’’’, d’’’). Blue fluorescence indicates DAPI staining of cell nuclei. Merged pictures are shown in the fourth column (d, d’, d’’, and d’’’). In control HRECs, green fluorescence is detectable only at nuclear level; an increase (indicating cPLA2 activation) can be observed in both cytoplasm and nuclei in HG-culture conditions, (b’’’ vs. b’ and b’’ vs. b, respectively). Panel (**E**): immunocytochemical staining for iPLA2 (green fluorescence) and β-actin (red fluorescence) in HRECs grown in normal glucose (NG, 5 mM; a, b, c, d), in normal glucose plus 80 ng/mL of VEGF-A (a’, b’, c’, d’), in high glucose (HG, 25 mM; a’’, b’’, c’’, d’’) or in high glucose supplemented with 80 ng/mL of VEGF-A (HG + VEGF-A; a’’’, b’’’, c’’’, d’’’). Blue fluorescence indicates DAPI staining of cell nuclei. Merged pictures are shown in the fourth column (d, d’, d’’, and d’’’). Immunostaining for iPLA2 is increased in HRECs cultured in high glucose supplemented with 80 ng/mL of VEGF-A (b’’’ vs. b’’, b’, and b). Magnification: ×40; scale bars: 100 µm.

Previous data suggested that (i) VEGF-A/PLA2 axis blockade could represent a promising strategy to prevent HG-induced endothelial cell damage and (ii) exogenous VEGF-A increases glucose toxicity in HRECs. Then, we tested the hypothesis that VEGF-A-mediated increase in HG toxicity may involve ERK1/2/PLA2 pathway activation in HREC. To this end, we analyzed protein levels of total ERK1/2, cPLA2, and iPLA2, as well as phospho-ERK1/2 and phospho-cPLA2 in HRECs treated with NG or HG with or without 80 ng/mL of VEGF-A for 48 h using both Western blot and immunocytochemical analysis. As reported in Figure 5 panels A and B, increased levels of phospho-ERK1/2 were observed in HRECs treated with VEGF-A (about 4 fold, *p* < 0.05) or HG (of about 3 fold, *p* < 0.05) in comparison to controls. Co-treatment with VEGF-A and HG triggered a further increase in phospho-ERK1/2 (*p* < 0.05). The different treatments did not change total ERK1/2 protein levels. Immunocytochemical analysis (Figure 5 panel C) was consistent with Western blot data, as indicated by enhanced immunoreactivity for phospho-ERK1/2 (green fluorescence, FITC) in HRECs stimulated with VEGF-A or HG (panel C images b’ and b’’ vs. b). Basal green fluorescence observed in NG-cultured cells was almost undetectable and mostly localized in the cytoplasm; in VEGF-A treated cells, green fluorescence was observed particularly in the nuclear region, consistent with previous reports [37]. Fluorescence intensity for phospho-ERK1/2 was markedly higher in HRECs treated with HG and VEGF-A in comparison to the other conditions and still localized in the nuclear region (panel C, image b’’’ vs. image b’’). No fluorescence intensity changes were observed for total ERK1/2 staining (red fluorescence, CY3) following all different treatments, indicating no changes in protein levels (panels C, images c-c’’’). 

Regarding PLA2, Western blot analysis showed enhanced phospho-cPLA2 levels by about 3-fold (*p* < 0.05) in HRECs co-treated with VEGF-A or HG, compared to controls (Figure 5 panels A and B). Increased phosphorylation in cPLA2 is suggestive of an enhanced enzymatic activity and thereby in arachidonic acid release. Total cPLA2 protein levels were unchanged in HRECs following treatments. Twenty-four-hour exposure to HG and VEGF-A induced the strongest increase (about 5-fold, *p* < 0.05) in phospho-cPLA2 (Figure 5 panels A and B). 

Stimulation of HREC with VEGF-A or HG did not changed iPLA2 protein levels, whereas treatment with HG and VEGF-A increased by about 2-fold the iPLA2 levels. 

Activation and subcellular localization of cPLA2 and iPLA2 were further investigated by immunofluorescence microscopy and data are shown in Figure 5 panels D and E, respectively. Intensity of green fluorescence (FITC) for phospho-cPLA2 staining was higher in HRECs treated with VEGF-A or HG in comparison to controls and was distributed mostly in the nuclear region (Figure 5 panel D, images b’ and b’’ vs. b). This different localization of phospho-cPLA2 staining is consequence of translocation events and suggestive of increased enzymatic activity. A further increase in phospho-cPLA2 immunoreactivity was observed in HRECs treated with HG and VEGF-A, still in the nuclear region (Figure 5 panel D, image b’’’ vs. image b-b’’). Total cPLA2 staining (red fluorescence, CY3) showed similar intensity for all treatment. Fluorescence intensity of iPLA2 staining was similar in HRECs treated with VEGF-A or HG compared to NG-cultured cell; however, we observed a higher immunoreactivity in iPLA2 of HRECs following treatment with HG and VEGF-A. Overall, these data indicate that, in HRECs, exogenous VEGF-A participates in an additive manner to HG, in activating ERK1/2/PLA axis.

### 2.6. Selective PLA2 Silencing Reduces the Detrimental Effects of HG in HRECs

**Figure 6:**
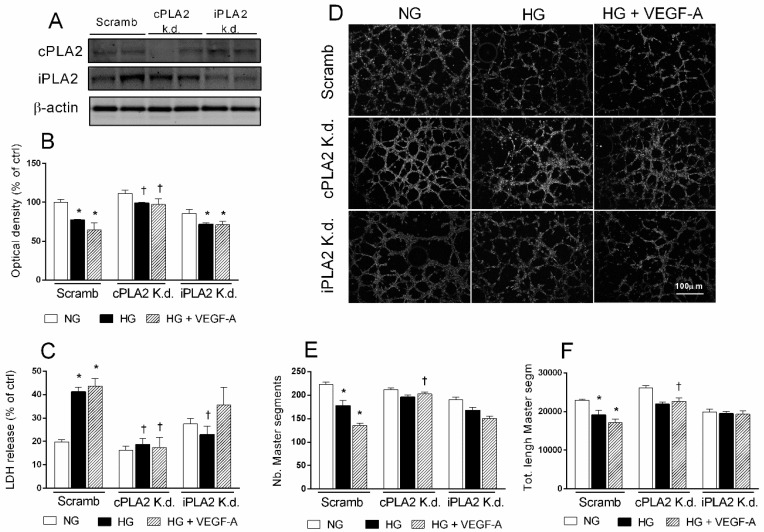
Effect of PLA2 silencing on detrimental effects of high glucose (HG) in human retinal endothelial cells (HRECs). Cell lysates of HRECs transfected with siRNA against cPLA2 (cPLA2 K.d.), iPLA2 (iPLA2 K.d.), and scrambled siRNA (Scramb) were analyzed by Western blot 48 h after transfection using antibodies against total cPLA2 and iPLA2 to evaluate efficiency of knockdown. Western blot analysis of lysates from two randomly selected independent transfections are shown in panel (**A**). Immunoblotting of β-actin was used to assess equal loading (30 µg of protein per lane). Transfected HRECs were incubated with normal glucose (5 mM, NG), high glucose (25 mM HG), or HG plus VEGF-A (80 ng/mL) for 48 h and analyzed in MTT assay (panel **B**) or LDH release assay (panel **C**). Transfected cells were also seeded into 96-well plate coated with matrigel at a density of 1.5 × 10^4^ and incubated as described above. After 16 h, tube like-structures were photographed (panel **D**) and images were analyzed for total number (panel **E**) and length (panel **F**) of tube-like structures with Image J software. Values are expressed as a mean ± SEM of three independent experiments, each run in triplicate. * *p* < 0.05 vs. scrambled NG; † *p* < 0.05 vs. scramb HG or scramb HG plus VEGF-A. One-way ANOVA, followed by Tukey’s test.

To further address the involvement of PLA2 in damage induced by HG and/or VEGF-A in human retinal endothelial cells, we silenced PLA2 by using small interfering RNAs. Specificity and yield of transient cPLA2 and iPLA2 knockdown (k.d.) was revealed by Western blot analysis of lysates obtained from different preparations of HRECs, after 48 h from transfection with specific siRNAs. As shown in Figure 6 panel A, protein basal levels of both cPLA2 and iPLA2 were strongly attenuated in HRECs transfected with 50 nmol/mL of siRNA, compared with cells transfected with scramble siRNA. After transfection protocol, cells were treated with NG, HG, or HG and VEGF-A for 48 h. In HRECs transfected with scramble siRNA, treatment with HG or HG and VEGF-A decreased cell viability (about 25% and 40%, respectively), as shown above in non-transfected cells. Specific cPLA2 knockdown significantly (*p* < 0.05) prevented the reduction of cell viability induced by either HG or HG and VEGF-A (Figure 6 panel B); such an effect was less pronounced with iPLA2 siRNA. These data were confirmed by LDH release, a cell membrane permeability assay. In HRECs transfected with scramble siRNA, HG, or HG and VEGF-A increased by about 2-fold (*p* < 0.05) LDH release (Figure 6 panel C). Transfection of cPLA2 siRNA prevented LDH release in both HG- and HG and VEGF-A treated cells. Knockdown of iPLA2, showed only a partial effect. Tube formation assay showed that either HG or HG and VEGF-A reduced tube formation in scramble siRNA transfected HRECs (Figure 6 panel D). In particular, analysis of images indicated that HG decreased number (by about 20%, *p* < 0.05) and length (by about 15%, *p* < 0.05) of master segments, while co-treatment with HG and VEGF-A decreased number (by about 40%. *p* < 0.05) and length (by about 25%, *p* < 0.05) of master segments (Figure 6 panels E and F).

Silencing of cPLA2 did not significantly affect tube formation in presence of NG; however, it prevented the reduction of both number and length in tube-like structure of HRECs induced by HG or HG and VEGF-A. Transfection with iPLA2 siRNA produced a mild, partial protection against cell injury mediated by HG or HG and VEGF-A. These data suggest that PLA2 activation, particularly cPLA2, mediates HG- and/or VEGF-A-induced cell damage in HRECs in vitro.

## 3. Discussion

Pro-inflammatory events are pivotal in the development of diabetic retinopathy, determining structural and functional alterations of retinal capillaries, starting from the early stages of retinopathy [39]. Dysregulation in PLA2s are involved in the progression of diabetic retinopathy, producing retinal alterations in phospholipid breakdown and arachidonic acid availability in endothelial cells and pericytes [40,41]. Increased production of PGE2 arises from COX-2 activity and contributes to cellular dysfunctions induced by diabetic conditions [18,19,42]; on the other hand, inhibition of COX-2 prevents the glucose-induced upregulation of retinal VEGF-A, decreases retinal vessel permeability, leukostasis, and cell death [43,44,45,46]. Furthermore, lysophosphatidylcholine (LPC), an enzymatic product of Lp-PLA2, increases vascular permeability during diabetic retinopathy, producing endothelial damage via VEGFR2 [17]. 

Here, we show that chemical blockade of cPLA2 through the selective inhibitor AACOCF3 protected human retinal endothelial cells against glucose-induced damage and restored viability and tube formation capability. This observation supports the idea that cPLA2 plays a pivotal role in the progression of DR since it mediates the HG-induced early damage of retinal endothelium and the diabetes-induced upregulation of retinal VEGF-A [44]. Furthermore, our findings indicate that increased phosphorylation of cPLA2 is linked to HG-induced VEGF-A upregulation; in fact, VEGF-A blockade with Aflibercept was able to prevent both HG-induced cell damage as well as phosphorylation of ERK1/2 and cPLA2. Our working hypothesis is that increased levels of VEGF-A induced by HG can exert an autocrine effect stimulating inflammatory processes, such as PLA2 activation, in retinal endothelial cell. Our data are also consistent with recent observations indicating that blockade of VEGF-A and/or PlGF by Aflibercept exerts protective effects on retinal cells, by inhibition of the ERK pathway and decreased expression of TNFα, in in vitro and in vivo models of DR [47]. VEGF-A is known to increase blood–retinal barrier permeability through the ubiquitin-mediated endocytosis of tight-junction induced by the PKCβ-mediated occludin phosphorylation [48,49], a mechanism directly involved in the disruption of blood–retinal barrier, responsible for edema and microhemorrhages. Here, we confirm that early up-regulation of VEGF-A induced by high concentration of glucose leads to an increased vascular dysfunction inducing inflammatory events [6]. Data reported in this work were produced in an in vitro model that mimics early phase of diabetic retinopathy as HRECs were subjected to a single treatment with HG for 48 h. In this context, we observed a modest albeit significant upregulation of VEGF-A, which did no considerably impact on cell proliferation. Chronic glucose treatment, instead, involves prolonged or fluctuating glucose exposure lasting several days and better mimic conditions of proliferative diabetic retinopathy involving further molecular mechanisms based on more robust VEGF-A upregulation or metabolic memory processes [18]. Advanced glycation end products (AGEs) plays a central role in the pathogenesis of DR and were found elevated in diabetes, since they originate from the reaction between a nucleophile, such as the amino group of a lysine, and a reducing sugars [50]. AGEs induce cell damage through binding to the AGE receptor (RAGE), expressed by different cells and comprised of three Ig domains that form its extracellular part [51,52]. Upon activation by its ligand, RAGE may trigger different and complex signaling pathways including the phosphatidylinositol 3-kinase (PI3K)/AKT pathway and the mitogen-activated protein kinases (MAPK) extracellular-signal-regulated kinases (ERK) 1/2 [53,54,55]. RAGE is involved in glucose-induced microvascular impairment by phosphorylation and translocation of NF-kB transcription factor that increase the expression of pro-inflammatory proteins such as tumor necrosis factor-α (TNF-α) and VEGF-A [8,56]. We have previously shown in HREC that RAGE specific stimulation by AGEs or HG produced the activation of ERK1/2/cPLA2 pathway, as revealed by the increased levels of their phosphorylation [29]. Here we show that exogenous VEGF-A produces a greater activation of ERK1/2/cPLA2 in HG compared to NG. Immunofluorescence staining suggests that these phosphorylated forms are preferentially associated with nuclear localization, presumably as a consequence of translocation events associated with increased enzymatic activity [57]. Moreover, exogenous VEGF-A enhanced glucose toxicity by further reducing cell viability and increasing cell damage (MTT and LDH release assays). Worthy of note, in our system, exogenous VEGF-A exerted the expected pro-angiogenic effects in the tube-formation assay, when tested in medium with normal glucose concentration; in contrast, in HG condition, exogenous VEGF-A lost its angiogenic effect, or at least it was superseded by its toxic effects. To interpret this paradoxical observation, we speculate that RAGE-mediated activation of the pro-inflammatory ERK1/2/cPLA2 axis can be significantly potentiated by the VEGF-A signaling, as indicated by increased phosphorylation levels in both ERK1/2 and cPLA2 following treatment with HG plus VEGF-A. However, we cannot exclude that in cells subjected to both HG and exogenous VEGF-A, an additional cPLA2 phosphorylation occurs at Ser^505^ by p38 MAP kinase, as described in different cell system [41,58,59,60]. In addition, in cells treated with both HG and exogenous VEGF-A we observed an upregulation of iPLA2 protein expression; the ensuing increase in enzymatic activity might further contribute to cell damage and/or impairment of angiogenic effects of VEGF-A. 

In our model, 80 ng/mL VEGF-A induced a moderate cellular effect with a diversified action based on conditions of HG or NG; considering that very high increase in VEGF-A levels have been reported in vitreous of patients with proliferative diabetic retinopathy [61], higher amounts of VEGF-A still need to be tested in this and/or other models, to precisely define the concentration range needed to activate aberrant cell proliferation or angiogenesis and the underlying molecular pathways. Finally, we found that selective inhibition of cPLA2 with specific siRNA strongly prevented HG-mediated dysfunction of HREC, even in the presence of exogenous VEGF-A, restoring cell viability and angiogenic capability, while selective iPLA2 siRNA exerted a milder protective effect. Worthy of note, these observations are entirely consistent with the results following specific chemical blockade of cPLA2 by AACOCF3 or iPLA2 by BEL. Great effort has been dedicated in developing synthetic inhibitors of PLA2 in order to verify their potential as medicinal agents and/or as tools to study the role of PLA2 isoforms [62]. Based on our data, we may anticipate that specific cPLA2 would afford a significant protection of endothelial cells in diabetic retinopathy; thus, new approaches of medicinal chemistry are desirable to generate specific cPLA2 inhibitors as new agents to treat diabetic retinopathy. 

## 4. Materials and Methods 

### 4.1. Reagents

Mouse monoclonal antibody against cPLA2 (catalog n. sc-454) and mouse monoclonal p44/42 MAPK (Erk1/2, catalog sc-135900) were purchased from Santa Cruz Biotechnology (Santa Cruz, CA, USA). Rabbit polyclonal antibody against phospho-cPLA2 (catalog n. 2831S), phospho-p44/42 MAPK (phospho-Erk1/2, catalog n. 9101) were purchased from Cell Signaling Technology (Danvers, MA, USA); rabbit polyclonal antibody against iPLA2 (catalog n. ab23706) and mouse monoclonal antibody against β-actin (catalog n. ab8226) were purchased from Abcam (Cambridge, UK). Secondary goat anti-rabbit IRDye 680 conjugated antibody (catalog n. 926-32221) and secondary goat anti-mouse IRDye 800 conjugated antibody (catalog n. 926-32210) were purchased from LI-COR Biosciences, (Lincoln, NE, USA). VEGF-A was from Peprotech (Rocky Hill, NJ, USA). Arachidonoyl trifluoro-methyl ketone (AACOCF3), a cPLA2 inhibitor, and iPLA2 inhibitor, bromoenol lactone (BEL) were purchased from Calbiochem (La Jolla, CA, USA). Aflibercept (Eylea^®^) was from Bayer Pharma (Berlin, Germany). Media, antibiotics, and other reagents for cell cultures were from Invitrogen Thermo Fisher Scientific (Monza, Italy).

### 4.2. Cell Cultures and Treatments

Primary human retinal endothelial cells (HRECs) were purchased from Innoprot (Elexalde Derio, Spain) and were fed with culture endothelial cell medium ECM, supplemented with 5% fetal bovine serum (FBS), 1% endothelial cell growth supplement (ECGS), 100 U/mL penicillin, and 100µg/mL streptomycin, provided by Innoprot. HRECs were characterized by their positive immunostaining for von Willebrand factor [63]. Cells were seeded in flasks or dishes pre-coated with a poly-L-Lysine solution (PLL), at a concentration of 1 mg/mL (Innoprot), for 1 h at 37 °C in a humidified atmosphere of 5% CO2. After removing the PLL solution, flasks and dishes were rinsed twice with sterile water. Before treatments, HRECs were starved with 1% FBS supplemented ECM for 4 h before treatments, in order to exclude serum unspecific effects on phosphorylation of protein. Then, cells were treated with 5 mM glucose (normal glucose or NG, control), 25 mM glucose (high glucose, HG), or 25 mM mannitol (high mannitol, HM) in 1% FBS ECM medium for 48 h. Control medium (NG), HM or HG medium were supplemented with VEGF-A (40 and 80 ng/mL, VEGF-A called “exogenous”), PLA2 inhibitor AACOCF3 (0.5, 3, and 15 µM), iPLA2 inhibitor BEL (0.5, 1, and 5 µM), or Aflibercept (1, 5, and 40 µg/mL), as appropriate to each experimental condition. Cells reached about 70% confluence prior to the addition of treatments and were used at passage between P3 and P9. After treatment, cells were washed twice in PBS and subjected to subsequent analyses.

### 4.3. Cell Viability Assay

Cell viability was determined by the 3-[4,5-dimethylthiazol-2-y l]-2,5-diphenyl tetrasodium bromide (MTT assay, Chemicon, Temecula, CA, USA). HRECs were seeded at 1.5 × 10^4^ cells per well into 96-well plates. After overnight growth, cells were incubated with different treatments, as appropriate. At the end of treatment, 20 μL of 5 mg/mL MTT was added to the medium and was incubated for 4 h at 37 °C. The supernatant was removed and 150 μL of DMSO used to dissolve the precipitate. Absorbance of mixtures was determined at 570 nm in a plate reader (VariosKan, Thermo Fisher Scientific, Waltham, MA, USA). 

### 4.4. Lactate Dehydrogenase (LDH) Release

LDH activity was tested in culture supernatants and was considered as biomarker of cell injury related to the permeability of cell membranes. We detected LDH activity in using a commercial LDH Activity Assay kit (Roche Diagnostics, Indianapolis, IN, USA) and expressed in % [(sample absorbance/lysed cell absorbance-control absorbance) × 100]. 

### 4.5. Immunocytochemical Analysis

HRECs were seeded in pre-coated glass chamber slides pre-treated with PLL. After incubation period and treatments, cells were washed with PBS, fixed for 30 min at 4 °C with paraformaldehyde (4% in PBS) and further incubated for 30 min in PBS, containing normal goat serum (5%) and triton (0.1%). Subsequently, cells were incubated overnight at 4 °C with primary antibodies (in PBS/triton 0.1%). Antibody concentrations were: mouse anti cPLA2 (Santa Cruz Biotechnology) 1:100, rabbit anti phospho-cPLA2 (Cell Signaling Technology) 1:90, rabbit anti iPLA2 1:120, mouse anti β-actin 1:100, mouse anti ERK1/2 1:120, and rabbit anti phospho-ERK1/2 1:90. Then, cells were washed three times with 0.1% Tween 20 in PBS and were incubated for 1 h at room temperature with secondary antibodies, FITC-conjugated goat anti-rabbit (1:300 dilution, Life Technologies) or Cy3-conjugated goat anti-mouse (Abcam) in the dark. Control samples were carried out by omitting the primary or secondary antibodies in order to assess the specificity of immunostaining. Subsequently, cells were washed with 0.1% Tween 20 in PBS for three times, nuclei were stained for 10 min with 4′,6-diamidino-2-phenylindole (DAPI, 1:10,000, Life Technologies), in the dark, at room temperature. Slides were then mounted with mounting medium (Life Technologies) and analyzed with an inverted fluorescence microscope (Leica, Wetzlar, Germany) equipped with a computer assisted digital camera (Nikon, Carl Zeiss, Oberkochen, Germany). Fluorescence was acquired separately with FITC and CY3 filters to acquire the anti-rabbit (green fluorescence) and anti-mouse (red fluorescence)-conjugated signals respectively. Nuclei were highlighted with UV light and merges of the three images was also reported. Images were acquired by applying the same parameters (time of exposition and fluorescence intensity) throughout all the acquisitions. 

### 4.6. Tube Formation Assay

Tube formation assay was performed in vitro with Matrigel Basement Membrane Matrix system (Becton and Dickinson, Milano, Italy). The experimental protocol was run according to the manufacturer’s instructions in 96-well plates. In brief, gel solution was thawed at 4 °C overnight, then appropriate numbers of wells were coated with 50 μL of Matrigel and allowed to solidify at 37 °C for 2 h. HRECs were seeded at density of 15 × 10^3^ cells per well in 100 μL of assay medium, containing HM, HG with or without Aflibercept (40 μg/mL), AACOCF3 (15 μM) or BEL (5 μM). Each condition was run in triplicate. Matrigel provided a three-dimensional support that allowed the formation of a tube-like structure network suggestive of in vivo capillaries. After 5 h of incubation, tube-like structures were photographed by using an inverted microscope. Number and tube length of the master segments were quantified with the Image J software (NIH, Bethesda, MD, USA). 

### 4.7. Western Blot Analysis

After treatment, HRECs were lysed with RIPA Buffer (with protease and phosphatase inhibitors cocktail) (Sigma-Aldrich, St. Louis, MO, USA). Extracted proteins from whole lysates were quantified by Bradford assay (Sigma-Aldrich) and 40 µg were loaded on precast polyacrylamide NuPageTM 10% Bis-Tris Gels, run in SDS-PAGE and after transfer to nitrocellulose membranes. Immunoblot was preceded by 30 min incubation with Odyssey Blocking Buffer (LI-COR Biosciences, Lincoln, NE, USA) to membranes and subsequently, membranes were incubated at 4 °C overnight with antibodies against target proteins in blocking buffer. The dilutions were: anti cPLA2 1:700, anti phospho-cPLA2 1:800, anti ERK1/2 1:800, anti phospho-ERk1/2 1:400, anti iPLA2 1:500, and anti β-actin 1:1000. β-actin blotting was used as the loading control. After washing, membranes were incubated with secondary fluorescent antibodies (1:20,000 dilution) for 1 h at room temperature and immunoblots were detected through Odyssey imaging system (LI-COR). Densitometry analyses of blots were performed using the Image J software. 

### 4.8. Transfection of siRNA

HRECs were treated with pre-designed siRNA duplex obtained from Ambion (Chicago, IL, USA). Two sets of oligonucleotides were used: set 1 directed to iPLA2 (Gene Bank NM_001004426) with sequence 5′ -GGUGUGAGAUGGUCGGUAU-3′, 5′-ACGCUGAGAUGGCCCGAAU-3′, 5′-CAGCAAGGAUCCUCGCUAU-3′, and 5′-CAGAAUGCUUCCAAUCGUA-3′. Set 2 against cPLA2 (Gene Bank NM_001311193) with sequence 5′-AGAAUUUAGUCCAAUCGAA-3′, 5′-CAUAUCUACACAUGCGAAA-3′, 5′-CAGAUGAAUUUGAACGAAU-3′, and 5′-GCGGAAAGAGAGUACCAAA-3′. The control siRNA was a pool of scrambled, non-targeting siRNA (Cat. AM4641, Ambion). Transfections (50 nmol/mL siRNAs) were carried out by INTERFERinTM reagent (Ambion) according to the manufacturer’s instructions. The efficiency was assessed by siGLO green transfection indicator (Dharmacon, Lafayette, CO, USA). Immunoblots eventually confirmed the reduction of the target protein. The effects of gene silencing on glucose treatments were tested 48 h after transfection.

### 4.9. Real-Time Reverse Transcriptase-Polymerase Chain Reaction (RT-qPCR)

Cells were lysed and total RNA extracted by TRIzol (Invitrogen, Thermo Fisher Scientific). RNA concentration and purity were assessed by measuring optical density at 260 and 280 nm. First-strand cDNA synthesis was carried out at 50 °C for 50 min by reverse transcription (RT) of 1 µg RNA in a 20 μL reaction volume, containing 200 U SuperScript III, 50 ng random hexamers, 1.25 mM dNTP, 10 mM dithiothreitol, 50 mM Tris-HCl, pH 8.3, 75 mM KCl, 3 mM MgCl2 (Invitrogen, Thermo Fisher Scientific). The reaction was then stopped at 85 °C for 5 min. Aliquots of 5 ng cDNA were amplified for forty amplification cycles by using Quant Studio 3 (Applied Biosystems, Thermo Fisher Scientific, Waltham, MA, USA). Each PCR reaction contained 0.8 μM forward and reverse specific primers, 1X iTaq™ Universal SYBR^®^ Green Supermix (Bio-Rad Laboratories, Milan, Italy) and 1 µL cDNA, in 10 μL final volume. Results were normalized by 18S ribosomal RNA and analyzed by the ΔΔCt method. Primers were from Eurofin Genomics (Milan, Italy). VEGF-A (Acc. Number: NM_001025366.3) was amplified with primer Forward 5′-ATCTTCAAGCCATCCTGTGTGC-3′ and Reverse 5′-GAGGTTTGATCCGCATAATCTG-3′, producing an amplicon of 121 bp. Reference housekeeping gene 18S rRNA (Acc. Number: NR_146119) was amplified with primer Forward 5′-TAAGTCCCTGCCCTTTGTACACA-3′ and Reverse 5′-GATCCGAGGGCCTCACTAAAC-3′ producing an amplicon of 69 bp. The specificity of the PCR reaction was assessed by the denaturation temperature of the amplification products [64].

### 4.10. Statistical Analysis

Data are illustrated as mean ± SEM. The different groups/conditions were compared by one-way analysis of variance (ANOVA) and Tukey–Kramer post hoc test; statistical significance was set at *p*-value < 0.05.

## 5. Conclusions

In vitro models of DR differ on the basis of the concentration and duration of high glucose treatment. A single glucose treatment at a concentration of 25 mM for 48 h mimics retinal endothelial cellular insult that occurs in the early stages of DR. In this work, we observed that HG-induced toxicity is mediated by the activation of ERK1/2/cPLA axis in HREC. The activation of the PLA2, and in particular cPLA2, represents the major rate limiting step of the retinal endothelium in the production of arachidonic acid and the downstream lipid mediators of inflammation. Interestingly, we observed that exogenous VEGF-A potentiated the effect of HG in the activation of ERK1/2/cPLA2 pathway and iPLA2. As consequence, treatment of HREC with HG supplemented with VEGF-A increased cell damage and further reduced tube formation potential. Thus, in pro-inflammatory condition induced by HG, low levels of VEGF-A represent a harmful stimulus for retinal endothelial cells, rather than an angiogenesis stimulating factor.

## References

[1] Ma¨kimattila S., Virkama¨ki A., Groop P.-H., Cockcroft J., Utriainen T., Fagerudd J., Yki-Ja¨rvinen H. (1996). Chronic Hyperglycemia Impairs Endothelial Function and Insulin Sensitivity Via Different Mechanisms in Insulin-Dependent Diabetes Mellitus. Circulation.

[2] Chou J., Rollins S., Fawzi A.A. (2014). Role of endothelial cell and pericyte dysfunction in diabetic retinopathy: Review of techniques in rodent models. Single Molecule and Single Cell Sequencing.

[3] Wang W., Lo A.C.Y. (2018). Diabetic Retinopathy: Pathophysiology and Treatments. Int. J. Mol. Sci..

[4] Brownlee M. (2005). The Pathobiology of Diabetic Complications: A Unifying Mechanism. Diabetes.

[5] Kowluru R.A., Chan P.-S. (2007). Oxidative Stress and Diabetic Retinopathy. Exp. Diabetes Res..

[6] Antonetti D.A., Klein R., Gardner T.W. (2012). Diabetic Retinopathy. N. Engl. J. Med..

[7] Pusparajah P., Elee L.-H., Kadir K.E. (2016). Molecular Markers of Diabetic Retinopathy: Potential Screening Tool of the Future?. Front. Physiol..

[8] Tang J., Kern T.S. (2011). Inflammation in diabetic retinopathy. Prog. Retin. Eye Res..

[9] Cummings B.S., McHowat J., Schnellmann R.G. (2000). Phospholipase A(2)s in cell injury and death. J. Pharmacol. Exp. Ther..

[10] Glaser K.B. (1995). Regulation of Phospholipase A2 Enzymes: Selective Inhibitors and their Pharmacological Potential. Advances in Pharmacology.

[11] Wu X., Walker C.L., Lu Q., Wu W., Eddelman D.B., Parish J.M., Xu X.-M. (2016). RhoA/Rho Kinase Mediates Neuronal Death Through Regulating cPLA2 Activation. Mol. Neurobiol..

[12] Rosenson R.S., Hurt-Camejo E. (2012). Phospholipase A2 enzymes and the risk of atherosclerosis. Eur. Hear. J..

[13] Sun G.Y., Chuang D.Y., Zong Y., Jiang J., Lee J.C.M., Gu Z., Simonyi A. (2014). Role of Cytosolic Phospholipase A2 in Oxidative and Inflammatory Signaling Pathways in Different Cell Types in the Central Nervous System. Mol. Neurobiol..

[14] Taketo M.M., Sonoshita M. (2002). Phospholipase A2 and apoptosis. Biochim. Biophys. Acta.

[15] Chang J.F., Yeh J.C., Ho C.T., Liu S.H., Hsieh C.Y., Wang T.M., Chang S.W., Lee I.T.K., Huang Y., Wang J.Y. (2019). Targeting ROS and cPLA2/COX2 Expressions Ameliorated Renal Damage in Obese Mice with Endotoxemia. Int. J. Mol. Sci..

[16] Gong Y., Jin X., Wang Q.-S., Wei S.-H., Hou B.-K., Li H.-Y., Zhang M.-N., Li Z.-H. (2014). The involvement of high mobility group 1 cytokine and phospholipases A2 in diabetic retinopathy. Lipids Health Dis..

[17] Canning P., Kenny B.-A., Prise V., Glenn J., Sarker M.H., Hudson N., Brandt M., Lopez F.J., Gale D., Luthert P.J. (2016). Lipoprotein-associated phospholipase A2 (Lp-PLA2) as a therapeutic target to prevent retinal vasopermeability during diabetes. Proc. Natl. Acad. Sci. USA.

[18] Lupo G., Motta C., Giurdanella G., Anfuso C.D., Alberghina M., Drago F., Salomone S., Bucolo C. (2013). Role of phospholipases A2 in diabetic retinopathy: In vitro and in vivo studies. Biochem. Pharmacol..

[19] Giurdanella G., Anfuso C.D., Olivieri M., Lupo G., Caporarello N., Eandi C.M., Drago F., Bucolo C., Esalomone S. (2015). Aflibercept, bevacizumab and ranibizumab prevent glucose-induced damage in human retinal pericytes in vitro, through a PLA2/COX-2/VEGF-A pathway. Biochem. Pharmacol..

[20] Balsinde J., Balboa M.A., Insel P.A., Dennis E.A. (1999). Regulation and inhibition of phospholipase A2. Annu. Rev. Pharmacol. Toxicol..

[21] Murakami M., Kudo I. (2002). Phospholipase A2. J. Biochem..

[22] Leslie C.C. (2015). Cytosolic phospholipase A2: Physiological function and role in disease. J. Lipid Res..

[23] Ma Z., Turk J. (2001). The molecular biology of the group VIA Ca2+-independent phospholipase A2. Prog. Nucleic Acid Res. Mol. Biol..

[24] Balsinde J., Balboa M.A. (2005). Cellular regulation and proposed biological functions of group VIA calcium-independent phospholipase A in activated cells. Cell. Signal..

[25] Ramanadham S., Ali T., Ashley J.W., Bone R.N., Hancock W.D., Lei X. (2015). Calcium-independent phospholipases A2and their roles in biological processes and diseases. J. Lipid Res..

[26] Lei X., Zhang S., Barbour S.E., Bohrer A., Ford E.L., Koizumi A., Papa F.R., Ramanadham S. (2010). Spontaneous development of endoplasmic reticulum stress that can lead to diabetes mellitus is associated with higher calcium-independent phospholipase A2 expression: A role for regulation by SREBP-1. J. Biol. Chem..

[27] Veret J., Coant N., Berdyshev E.V., Skobeleva A., Therville N., Bailbé D., Gorshkova I., Natarajan V., Portha B., Le Stunff H. (2011). Ceramide synthase 4 and de novo production of ceramides with specific N-acyl chain lengths are involved in glucolipotoxicity-induced apoptosis of INS-1 β-cells. Biochem. J..

[28] Boslem E., MacIntosh G., Preston A.M., Bartley C., Busch A.K., Fuller M., Laybutt D.R., Meikle P.J., Biden T.J. (2011). A lipidomic screen of palmitate-treated MIN6 beta-cells links sphingolipid metabolites with endoplasmic reticulum (ER) stress and impaired protein trafficking. Biochem. J..

[29] Giurdanella G., Lazzara F., Caporarello N., Lupo G., Anfuso C.D., Eandi C.M., Leggio G.M., Drago F., Bucolo C., Salomone S. (2017). Sulodexide prevents activation of the PLA2/COX-2/VEGF inflammatory pathway in human retinal endothelial cells by blocking the effect of AGE/RAGE. Biochem. Pharmacol..

[30] Askarova S., Yang X., Sheng W., Sun G.Y., Lee J.C. (2011). Role of Aβ-receptor for advanced glycation endproducts interaction in oxidative stress and cytosolic phospholipase A₂ activation in astrocytes and cerebral endothelial cells. Neuroscience.

[31] Nicotra A., Lupo G., Giurdanella G., Anfuso C.D., Ragusa N., Tirolo C., Marchetti B., Alberghina M. (2005). MAPKs mediate the activation of cytosolic phospholipase A2 by amyloid beta(25-35) peptide in bovine retina pericytes. Biochim. Biophys. Acta.

[32] Zhang Q., Wang D., Singh N.K., Kundumani-Sridharan V., Gadiparthi L., Rao C.H.M., Rao G.N. (2011). Activation of Cytosolic Phospholipase A2 Downstream of the Src-Phospholipase D1 (PLD1)-Protein Kinase C γ (PKC γ) Signaling Axis Is Required for Hypoxia-Induced Pathological Retinal Angiogenesis. J. Biol. Chem..

[33] Singh N.K., Hansen D.E., Kundumani-Sridharan V., Rao G.N. (2013). Both Kdr and Flt1 play a vital role in hypoxia-induced Src-PLD1-PKCγ-cPLA2 activation and retinal neovascularization. Blood.

[34] Choudhuri S., Chowdhury I.H., Das S., Dutta D., Saha A., Sarkar R., Mandal L.K., Mukherjee S., Bhattacharya B. (2015). Role of NF-κB activation and VEGF gene polymorphisms in VEGF up regulation in non-proliferative and proliferative diabetic retinopathy. Mol. Cell. Biochem..

[35] Wheeler-Jones C., Abu-Ghazaleh R., Cospedal R., Houliston R.A., Martin J., Zachary I. (1997). Vascular endothelial growth factor stimulates prostacyclin production and activation of cytosolic phospholipase A2 in endothelial cells via p42/p44 mitogen-activated protein kinase. FEBS Lett..

[36] Toft-Kehler A.K., Andersen C., Andreasen J.R., Vohra R., Junker N., Poulsen K.A., Kolko M. (2012). Interaction between VEGF and Calcium-Independent Phospholipase A2in Proliferation and Migration of Retinal Pigment Epithelium. Curr. Eye Res..

[37] Giurdanella G., Motta C., Muriana S., Arena V., Anfuso C.D., Lupo G., Alberghina M. (2011). Cytosolic and calcium-independent phospholipase A2 mediate glioma-enhanced proangiogenic activity of brain endothelial cells. Microvasc. Res..

[38] Ito Y., Iwamoto Y., Tanaka K., Okuyama K., Sugioka Y. (1996). A quantitative assay using basament extracts to study tumor angiogensis in vivo. Int. J. Cancer.

[39] Kaul K., Hodgkinson A., Tarr J.M., Kohner E.M., Chibber R. (2010). Is Inflammation a Common Retinal-Renal-Nerve Pathogenic Link in Diabetes?. Curr. Diabetes Rev..

[40] Tikhonenko M., Lydic T.A., Wang Y., Chen W., Opreanu M., Sochacki A., McSorley K.M., Renis R.L., Kern T., Jump D.B. (2009). Remodeling of Retinal Fatty Acids in an Animal Model of Diabetes: A Decrease in Long-Chain Polyunsaturated Fatty Acids Is Associated with a Decrease in Fatty Acid Elongases Elovl2 and Elovl4. Diabetes.

[41] Barnett J.M., Mccollum G.W., Penn J.S. (2010). Role of Cytosolic Phospholipase A2 in Retinal Neovascularization. Investig. Opthalmol. Vis. Sci..

[42] Abu El-Asrar A.M., Missotten L., Geboes K. (2008). Expression of cyclo-oxygenase-2 and downstream enzymes in diabetic fibrovascular epiretinal membranes. Br. J. Ophthalmol..

[43] Joussen A.M., Poulaki V., Mitsiades N., Kirchhof B., Koizumi K., Döhmen S., Adamis A.P. (2002). Nonsteroidal anti-inflammatory drugs prevent early diabetic retinopathy via TNF-α suppression. FASEB J..

[44] Ayalasomayajula S.P., Kompella U.B. (2003). Celecoxib, a selective cyclooxygenase-2 inhibitor, inhibits retinal vascular endothelial growth factor expression and vascular leakage in a streptozotocin-induced diabetic rat model. Eur. J. Pharmacol..

[45] Brust A.-K., Ulbrich H.K., Seigel G.M., Pfeiffer N., Grus F.H. (2008). Effects of Cyclooxygenase Inhibitors on Apoptotic Neuroretinal Cells. Biomark. Insights.

[46] Radi Z.A., Render J.A. (2008). The pathophysiologic role of cyclo-oxygenases in the eye. J. Ocul. Pharmacol. Ther..

[47] Lazzara F., Fidilio A., Platania C.B.M., Giurdanella G., Salomone S., Leggio G.M., Tarallo V., Cicatiello V., De Falco S., Eandi C.M. (2019). Aflibercept regulates retinal inflammation elicited by high glucose via the PlGF/ERK pathway. Biochem. Pharmacol..

[48] Murakami T., Felinski E.A., Antonetti D.A. (2009). Occludin Phosphorylation and Ubiquitination Regulate Tight Junction Trafficking and Vascular Endothelial Growth Factor-induced Permeability. J. Biol. Chem..

[49] Harhaj N.S., Felinski E.A., Wolpert E.B., Sundstrom J.M., Gardner T.W., Antonetti D.A. (2006). VEGF Activation of Protein Kinase C Stimulates Occludin Phosphorylation and Contributes to Endothelial Permeability. Investig. Opthalmol. Vis. Sci..

[50] Thornalley P.J., Battah S., Ahmed N., Karachalias N., Agalou S., Babaei-Jadidi R., Dawnay A. (2003). Quantitative screening of advanced glycation endproducts in cellular and extracellular proteins by tandem mass spectrometry. Biochem. J..

[51] Teissier T., Boulanger É. (2019). The receptor for advanced glycation end-products (RAGE) is an important pattern recognition receptor (PRR) for inflammaging. Biogerontology.

[52] Kandarakis S.A., Piperi C., Topouzis F., Papavassiliou A.G. (2014). Emerging role of advanced glycation-end products (AGEs) in the pathobiology of eye diseases. Prog. Retin. Eye Res..

[53] Liu Y., Liang C., Liu X., Liao B., Pan X., Ren Y., Fan M., Li M., He Z., Wu J. (2010). AGEs increased migration and inflammatory responses of adventitial fibroblasts via RAGE, MAPK and NF-κB pathways. Atherosclerosis.

[54] Schiekofer1 S., Andrassy1 M., Chen J., Rudofsky G., Schneider J., Wendt T., Stefan N., Humpert P., Fritsche A., Stumvoll M. (2003). Acute Hyperglycemia Causes Intracellular Formation of CML and Activation of ras, p42/44 MAPK, and Nuclear Factor κB in PBMCs. Diabetes.

[55] Yeh C.H., Sturgis L., Haidacher J., Zhang X.N., Sherwood S.J., Bjercke R.J., Juhasz O., Crow M.T., Tilton R.G., Denner L. (2001). Requirement for p38 and p44/p42 mitogen-activated protein kinases in RAGE-mediated nuclear factorkappaB transcriptional activation and cytokine secretion. Diabetes.

[56] Goldin A., Beckman J.A., Schmidt A.M., Creager M.A. (2006). Advanced Glycation End Products Sparking the Development of Diabetic Vascular Injury. Circulation.

[57] Anfuso C.D., Giurdanella G., Motta C., Muriana S., Lupo G., Ragusa N., Alberghina M. (2009). PKCalpha-MAPK/ERK-phospholipase A2 signaling is required for human melanoma-enhanced brain endothelial cell proliferation and motility. Microvasc. Res..

[58] Gijón M.A., Spencer D.M., Siddiqi A.R., Bonventre J.V., Leslie C.C. (2000). Cytosolic Phospholipase A2Is Required for Macrophage Arachidonic Acid Release by Agonists That Do and Do Not Mobilize Calcium. J. Biol. Chem..

[59] Fatima S., Khandekar Z., Parmentier J.H., Malik K.U. (2001). Cytosolic phospholipase A2 activation by the p38 kinase inhibitor SB203580 in rabbit aortic smooth muscle cells. J. Pharmacol. Exp. Ther..

[60] Mather A., Chen X.M., McGinn S., Field M.J., Summual S., Mangiafico S., Khang Y., Kelly D.J., Pollock C.A. (2009). High glucose induced endothelial cell growth inhibition is associated with an increase in TGFbeta1 secretion and inhibition of Ras prenylation via suppression of the mevalonate pathway. Int. J. Biochem. Cell Biol..

[61] Hernandez C., Lecube A., Segura R.M., Sararols L., Simó R. (2002). Nitric oxide and vascular endothelial growth factor concentrations are increased but not related in vitreous fluid of patients with proliferative diabetic retinopathy. Diabet. Med..

[62] Nikolaou A., Kokotou M.G., Vasilakaki S., Kokotos G. (2019). Small-molecule inhibitors as potential therapeutics and as tools to understand the role of phospholipases A2. Biochim. Biophys. Acta (BBA) Mol. Cell Biol. Lipids.

[63] Giurdanella G., Montalbano G., Gennuso F., Brancati S., Furno D.L., Augello A., Bucolo C., Drago F., Esalomone S. (2018). Isolation, cultivation, and characterization of primary bovine cochlear pericytes: A new in vitro model of stria vascularis. J. Cell. Physiol..

[64] Anfuso C.D., Longo A., Distefano A., Amorini A.M., Salmeri M., Zanghì G., Giallongo C., Giurdanella G., Lupo G. (2020). Uveal Melanoma Cells Elicit Retinal Pericyte Phenotypical and Biochemical Changes in an in Vitro Model of Coculture. Int. J. Mol. Sci..

